# Linear-time computation of minimal absent words using suffix array

**DOI:** 10.1186/s12859-014-0388-9

**Published:** 2014-12-20

**Authors:** Carl Barton, Alice Heliou, Laurent Mouchard, Solon P Pissis

**Affiliations:** Department of Informatics, King’s College London, The Strand, WC2R 2LS, London, UK; Inria Saclay-Île de France, AMIB, Bâtiment Alan Turing, Palaiseau, France; Laboratoire d’Informatique de l’École Polytechnique (LIX), CNRS UMR 7161, Palaiseau, France; University of Rouen, LITIS EA 4108, TIBS, Rouen, France

**Keywords:** Absent words, Minimal absent words, Suffix array

## Abstract

**Background:**

An *absent word* of a word *y* of length *n* is a word that does not occur in *y*. It is a *minimal absent word* if all its proper factors occur in *y*. Minimal absent words have been computed in genomes of organisms from all domains of life; their computation also provides a fast alternative for measuring approximation in sequence comparison. There exists an $\mathcal {O}(n)$-time and $\mathcal {O}(n)$-space algorithm for computing all minimal absent words on a fixed-sized alphabet based on the construction of suffix automata (Crochemore et al., 1998). No implementation of this algorithm is publicly available. There also exists an $\mathcal {O}(n^{2})$-time and $\mathcal {O}(n)$-space algorithm for the same problem based on the construction of suffix arrays (Pinho et al., 2009). An implementation of this algorithm was also provided by the authors and is currently the fastest available.

**Results:**

Our contribution in this article is twofold: first, we bridge this unpleasant gap by presenting an $\mathcal {O}(n)$-time and $\mathcal {O}(n)$-space algorithm for computing all minimal absent words based on the construction of suffix arrays; and second, we provide the respective implementation of this algorithm. Experimental results, using real and synthetic data, show that this implementation outperforms the one by Pinho et al. The open-source code of our implementation is freely available at http://github.com/solonas13/maw.

**Conclusions:**

Classical notions for sequence comparison are increasingly being replaced by other similarity measures that refer to the composition of sequences in terms of their constituent patterns. One such measure is the minimal absent words. In this article, we present a new linear-time and linear-space algorithm for the computation of minimal absent words based on the suffix array.

## Background

Sequence comparison is an important step in many important tasks in bioinformatics. It is used in many applications; from phylogenies reconstruction to the reconstruction of genomes. Traditional techniques for measuring approximation in sequence comparison are based on the notions of distance or of similarity between sequences; and these are computed through sequence alignment techniques. An issue with using alignment techniques is that they are computationally expensive: they require quadratic time in the length of the sequences. Moreover, in molecular taxonomy and phylogeny, for instance, whole-genome alignment proves both computationally expensive and hardly significant. These observations have led to increased research into *alignment free* techniques for sequence comparison. A number of alignment free techniques have been proposed: in [1], a method based on the computation of the shortest unique factors of each sequence is proposed; other approaches estimate the number of mismatches per site based on the length of exact matches between pairs of sequences [2].

Thus standard notions are gradually being complemented (or even supplanted) by other measures that refer, implicitly or explicitly, to the composition of sequences in terms of their constituent patterns. One such measure is the notion of words absent in a sequence. A word is an *absent word* of some sequence if it does not occur in the sequence. These words represent a type of *negative information*: information about what does not occur in the sequence. Noting the words which do occur in one sequence but do not occur in another can be used to detect mutations or other biologically significant events.

Given a sequence of length *n*, the number of absent words of length at most *n* can be exponential in *n*, meaning that using all the absent words for sequence comparison is more expensive than alignments. However, the number of certain subsets of absent words is only linear in *n*. An absent word of a sequence is a *shortest absent word* if all words shorter than it do occur in the sequence. An $\mathcal {O}(mn)$-time algorithm for computing shortest absent words was presented in [3], where *m* is a user-specified threshold on the length of the shortest absent words. This was later improved by [4], who presented an $\mathcal {O}(n \log \log n)$-time algorithm for the same problem. This has been further improved and an $\mathcal {O}(n)$-time algorithm was presented in [5].

A *minimal absent word* of a sequence is an absent word whose proper factors all occur in the sequence. Notice that minimal absent words are a superset of shortest absent words [6]. An upper bound on the number of minimal absent words is $\mathcal {O}(\sigma n)$ [7,8], where *σ* is the size of the alphabet. This suggests that it may be possible to compare sequences in time proportional to their lengths, for a fixed-sized alphabet, instead of proportional to the product of their lengths [1]. Theory and some applications of minimal absent words can be found in [9].

Recently, there has been a number of biological studies on the significance of absent words. The most comprehensive study on the significance of absent words is probably [10]; in this, the authors suggest that the deficit of certain subsets of absent words in vertebrates may be explained by the hypermutability of the genome. It was later found in [11] that the compositional biases observed in [10] for vertebrates are not uniform throughout different sets of minimal absent words. Moreover, the analyses in [11] support the hypothesis of the inheritance of minimal absent words through a common ancestor, in addition to lineage-specific inheritance, only in vertebrates. In [12], the minimal absent words in four human genomes were computed, and it was shown that, as expected, intra-species variations in minimal absent words were lower than inter-species variations. Minimal absent words have also been used for phylogenies reconstruction [13].

From an algorithmic perspective, an $\mathcal {O}(n)$-time and $\mathcal {O}(n)$-space algorithm for computing all minimal absent words on a fixed-sized alphabet based on the construction of suffix automata was presented in [7]. An alternative $\mathcal {O}(n)$-time solution for finding minimal absent words of length at most *ℓ*, such that $\ell = \mathcal {O}(1)$, based on the construction of tries of bounded-length factors was presented in [13]. A drawback of these approaches, in practical terms, is that the construction of suffix automata (or of tries) may have a large memory footprint. Due to this, an important problem is to be able to compute the minimal absent words of a sequence without the use of data structures such as the suffix automaton. To this end, the computation of minimal absent words based on the construction of suffix arrays was considered in [6]; although fast in practice, the worst-case runtime of this algorithm is $\mathcal {O}(n^{2})$. Alternatively, one could make use of the succinct representations of the bidirectional BWT, recently presented in [14], to compute all minimal absent words in time $\mathcal {O}(n)$. However, an implementation of these representations was not made available by the authors; and it is also rather unlikely that such an implementation will outperform an $\mathcal {O}(n)$-time algorithm based on the construction of suffix arrays.

### Our contribution

In this article, we bridge this unpleasant gap by presenting the first $\mathcal {O}(n)$-time and $\mathcal {O}(n)$-space algorithm for computing all minimal absent words of a sequence of length *n* based on the construction of suffix arrays. In addition, we provide the respective implementation of this algorithm. This implementation is shown to be more efficient than existing tools, both in terms of speed and memory.

## Methods

### Definitions and notation

To provide an overview of our result and algorithm, we begin with a few definitions. Let *y*=*y*[ 0]*y*[ 1]..*y*[ *n*−1] be a *word* of *lengthn*=|*y*| over a finite ordered *alphabetΣ* of size $\sigma = |\Sigma |=\mathcal {O}(1)$. We denote by *y*[ *i*..*j*]=*y*[ *i*]..*y*[ *j*] the *factor* of *y* that starts at position *i* and ends at position *j* and by *ε* the *empty word*, word of length 0. We recall that a prefix of *y* is a factor that starts at position 0 (*y*[ 0..*j*]) and a suffix is a factor that ends at position *n*−1 (*y*[ *i*..*n*−1]), and that a factor of *y* is a *proper* factor if it is not the empty word or *y* itself.

Let *x* be a word of length 0<*m*≤*n*. We say that there exists an *occurrence* of *x* in *y*, or, more simply, that *xoccurs iny*, when *x* is a factor of *y*. Every occurrence of *x* can be characterised by a starting position in *y*. Thus we say that *x* occurs at the *starting positioni* in *y* when *x*=*y*[ *i*..*i*+*m*−1]. Opposingly, we say that the word *x* is an *absent word* of *y* if it does not occur in *y*. The absent word *x*, *m*≥2, of *y* is *minimal* if and only if all its proper factors occur in *y*.

We denote by SA the *suffix array* of *y*, that is the array of length *n* of the starting positions of all sorted suffixes of *y*, i.e. for all 1≤*r*<*n*, we have *y*[ SA[ *r*−1]..*n*−1]<*y*[SA[ *r*]..*n*−1] [15]. Let lcp (*r*,*s*) denote the length of the longest common prefix of the words *y*[SA[ *r*]..*n*−1] and *y*[SA[ *s*]..*n*−1], for all 0≤*r*,*s*<*n*, and 0 otherwise. We denote by LCP the *longest common prefix* array of *y* defined by LCP [ *r*]=lcp(*r*−1,*r*), for all 1≤*r*<*n*, and LCP [ 0]=0. The inverse iSA of the array SA is defined by iSA[ SA[ *r*]]=*r*, for all 0≤*r*<*n*. SA [16], iSA, and LCP [17] of *y* can be computed in time and space $\mathcal {O}(n)$.

In this article, we consider the following problem:

MINIMALABSENTWORDS**Input:** a word *y* on *Σ* of length *n***Output:** for every minimal absent word *x* of *y*, one tuple <*a*,(*i*,*j*)>, such that *x* is defined by *x*[ 0]=*a*, *a*∈*Σ*, and *x*[ 1..*m*−1]=*y*[ *i*..*j*], *m*≥2.

### Algorithm MAW

In this section, we present algorithm MAW, an $\mathcal {O}(n)$-time and $\mathcal {O}(n)$-space algorithm for finding all minimal absent words in a word of length *n* using arrays SA and LCP.

We first give an example and explain how we can characterise the minimal absent words; then we introduce how their computation can be done efficiently by using arrays SA and LCP. Finally, we present in detail the two main steps of the algorithm.

**Figure 1:**
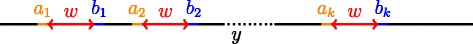
***k*** **occurrences of a factor** ***w*** **of** ***y*** **; they are preceded by** ***a***_***i***_ **and followed by** ***b***_***i***_ **.** If there exist *i*,*j*∈[ 1:*k*] such that (*a*_*i*_,*b*_*j*_)∉{(*a*_1_,*b*_1_),…,(*a*_*k*_,*b*_*k*_)} then *a*_*i*_ *w* *b*_*j*_ is a minimal absent word of *y*.

Intuitively, the idea is to look at the occurrences of a factor *w* of *y* and, in particular, at the letters that precede and follow these occurrences. If we find a couple (*a*,*b*), *a*,*b*∈*Σ*, such that *aw* and *wb* occur in *y*, but *awb* does not occur in *y*, then we can conclude that *awb* is a minimal absent word of *y*. For an illustration inspect Figure 1.

For example, let us consider the word *y*=AABABABB:

- factor *w*=AB occurs at:
  - position 1 preceded by A and followed by A
  - position 3 preceded by B and followed by A
  - position 5 preceded by B and followed by B
  We see that A*w* occurs and *w*B occurs as well but A*w*B does not occur in *y*, so AABB is a minimal absent word of *y*.
- factor *w*=BA occurs at:
  - position 2 preceded by A and followed by B
  - position 4 preceded by A and followed by B
  We cannot infer a minimal absent word.

**Figure 2:**
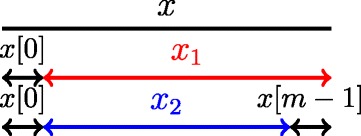
**A word** ***x*** **and its factors** ***x***_**1**_ **=** ***x*** **[ 1** ***.*** ***.*** ***m*** **−1] and** ***x***_**2**_ **=** ***x***_**1**_ **[0** ***.*** ***.*** **|** ***x***_**1**_ **|−2].**

A minimal absent word *x*[ 0..*m*−1] of a word *y*[ 0..*n*−1] is an absent word whose proper factors all occur in *y*. Among them, *x*_1_=*x*[ 1..*m*−1] and *x*_2_=*x*[ 1..*m*−2]=*x*_1_[ 0..|*x*_1_|−2] occur in *y* (inspect Figure 2); we will focus on these two factors to characterise the minimal absent words. To do so, we will consider each occurrence of *x*_1_ and *x*_2_, and construct the sets of letters that occur just before:

B_1_(*x*_1_) = {*y*[*j*−1]:*j* is the starting position of an occurrence of *x*_1_}

B_2_(*x*_1_) = {*y*[*j*−1]:*j* is the starting position of an occurrence of *x*_1_[0..|*x*_1_|−2]}.

#### **Lemma1**.

Let *x* and *y* be two words. Then *x* is a minimal absent word of *y* if and only if *x*[ 0] is an element of B_2_(*x*_1_) and not of B_1_(*x*_1_), with *x*_1_=*x*[ 1.. *m*−1].

#### *Proof*.

(⇒) Let *x*_1_ be a factor of *y*, *x*_2_ be the longest proper prefix of *x*_1_, and B_1_(*x*_1_) and B_2_(*x*_1_) the sets defined above. Further let *p* be a letter that is in B_2_(*x*_1_) but not in B_1_(*x*_1_). Then, there exists a starting position *j* of an occurrence of *x*_2_ such that *y*[ *j*−1]=*p*, so the word *px*_2_ occurs at position *j*−1 in *y*. *p* is not in B_1_(*x*_1_) so *px*_1_ does not occur in *x* and is therefore an absent word of *y*. *x*_1_ and *px*_2_ are factors of *y*, so all the proper factors of *px*_1_ occur in *y*, thus *px*_1_ is a minimal absent word of *y*.

(⇐) Let *x*[ 0..*m*−1] be a minimal absent word of *y*. Its longest proper prefix *x*[ 0..*m*−2]=*x*[ 0]*x*_1_[ 0..|*x*_1_|−2] occurs in *y*, so *x*[0] is in B_2_(*x*_1_). Its longest proper suffix, *x*_1_ occurs as well in *y*, but *x*=*x*[ 0]*x*_1_ is an absent word of *y* so it does not occur in *y* and *x*[ 0] is not in B_1_(*x*_1_).

#### **Lemma2**.

Let *x* be a minimal absent word of length *m* of word *y* of length *n*. Then there exists an integer *i*∈[ 0:*n*−1] such that *y*[ SA[ *i*]..SA[ *i*]+LCP[ *i*]]=*x*_1_ or *y*[ SA[ *i*]..SA[ *i*]+LCP[ *i*+1]]=*x*_1_, where *x*_1_=*x*[ 1..*m*−1].

#### *Proof*.

Let *j* be the starting position of an occurrence of *x*[ 0..*m*−2] in *y* and *k* the starting position of an occurrence of *x*_1_ in *y*. The suffixes *y*[ *j*+1..*n*−1] and *y*[ *k*..*n*−1] share *x*_2_=*x*[ 1..*m*−2] as a common prefix. As *x* is an absent word of *y*, this common prefix cannot be extended so *x*_2_ is the longest common prefix of those suffixes. By using iSA, the inverse suffix array, we have lcp(iSA[ *j*+1],iSA[ *k*])=*m*−2. Let us also note *s*_*k*_=iSA[ *k*] and *s*_*j*+1_=iSA[*j*+1]. We then have two possibilities:

- if *s*_*k*_>*s*_*j*+1_: for all *s* in [ *s*_*j*+1_+1:*s*_*k*_], we have LCP[ *s*]≥*m*−2, with equality holding for at least one position. Let us define *i*= max{*s*∈[ *s*_*j*+1_+1:*s*_*k*_]:LCP[ *s*]=*m*−2 }, the maximality of *i* implies that *i*=*s*_*k*_ or lcp(*i*,*s*_*k*_)>*m*−2 and thus, in both cases *y*[SA[ *i*]..SA[ *i*]+LCP[ *i*]]=*x*_1_.
- if *s*_*j*+1_>*s*_*k*_ : for all *s* in [ *s*_*k*_+1:*s*_*j*+1_], we have LCP[ *s*]≥*m*−2, with equality holding for at least one position. Let us define *i*= min{*s*∈[ *s*_*k*_:*s*_*j*+1_−1]:LCP[ *s*+1]=*m*−2 }, the minimality of *i* implies *i*=*s*_*k*_ or lcp(*s*_*k*_,*i*)>*m*−2 and thus, in both cases *y*[ SA[ *i*]..SA[ *i*]+LCP[ *i*+1]]=*x*_1_.

**Figure 3:**
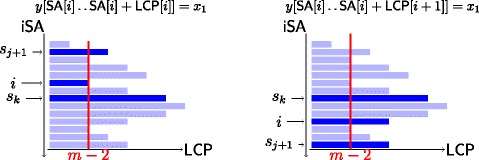
**Illustration of Lemma 2.**

For an illustration inspect Figure 3.

By Lemma 2, we can compute all minimal absent words of *y* by examining only the factors *S*_2*i*_=*y*[ SA[ *i*]..SA[ *i*]+LCP[ *i*]] and *S*_2*i*+1_=*y*[ SA[ *i*]..SA[ *i*]+LCP[ *i*+1]], for all *i* in [ 0:*n*−1]. We just need to construct the sets B_1_(*S*_2*i*_), B_2_(*S*_2*i*_) and B_1_(*S*_2*i*+1_), B_2_(*S*_2*i*+1_), where B_1_(*S*_*j*_) (resp. B_2_(*S*_*j*_)) is the set of letters that immediately precede an occurrence of the factor *S*_*j*_ (resp. the longest proper prefix of *S*_*j*_), for all *j* in [ 0:2*n*−1]. Then, by Lemma 1, the difference between B_2_(*S*_*j*_) and B_1_(*S*_*j*_), for all *j* in [ 0:2*n*−1], gives us all the minimal absent words of *y*.

Thus the important computational step is to compute these sets of letters efficiently. To do so, we visit twice arrays SA and LCP using another array denoted by B_1_ (resp. B_2_) to store set B_1_(*S*_*j*_) (resp. B_2_(*S*_*j*_)), for all *j* in [ 0:2*n*−1]. Both arrays B_1_ and B_2_ consist of 2*n* elements, where each element is a bit vector of length *σ*, the size of the alphabet, corresponding to one bit per alphabet letter. While iterating over arrays SA and LCP, we maintain another array denoted by Interval, such that, at the end of each iteration *i*, the *ℓ*^*th*^ element of Interval stores the set of letters we have encountered before the prefix of length *ℓ* of *y*[ SA[ *i*]..*n*−1]. Array Interval consists of max*i*∈ [0:*n*−1]LCP[ *i*]+1 elements, where each element is a bit vector of length *σ*.

During the first pass, we visit arrays SA and LCP from top to bottom. For each *i*∈[ 0:*n*−1], we store in positions 2*i* and 2*i*+1 of B_1_ (resp. B_2_) the set of letters that immediately precede occurrences of *S*_2*i*_ and *S*_2*i*+1_ (resp. their longest proper prefixes) whose starting positions appear before position *i* in SA. During the second pass, we go bottom up to complete the sets, which are already stored, with the letters preceding the occurrences whose starting positions appear after position *i* in SA. In order to be efficient, we will maintain a stack structure, denoted by LifoLCP, to store the LCP values of the factors that are prefixes of the one we are currently visiting.

#### Top-down pass

Each iteration of the top-down pass consists of two steps. In the first step, we visit LifoLCP from the top and for each LCP value read we set to zero the corresponding element of Interval; then we remove this value from the stack. We stop when we reach a value smaller or equal to LCP[ *i*]. We do this as the corresponding factors are not prefixes of *y*[ SA[ *i*]..*n*−1], nor will they be prefixes in the remaining suffixes. We push at most one value onto the stack LifoLCP per iteration, so, in total, there are *n* times we will set an element of Interval to zero. This step requires time and space $\mathcal {O}(n\sigma)$.

**Figure 4:**
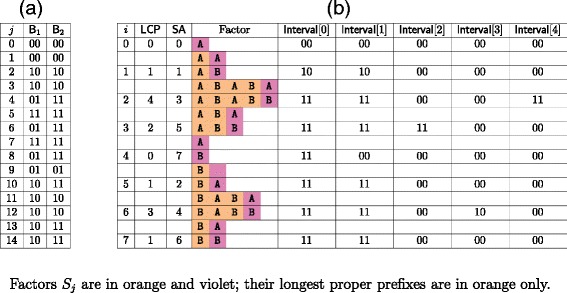
**Top-down pass.** **(a)** Arrays B_1_ and B_2_ obtained after the top-down pass for word *y*=AABABABB; **(b)** Elements of array Interval at the end of each iteration of the top-down pass.



For the second step, we update the elements that correspond to factors in the suffix array with an LCP value less than LCP[ *i*]. To do so, we visit the stack LifoLCP top-down and, for each LCP value read, we add the letter *y*[SA[ *i*]−1] to the corresponding element of Interval until we reach a value whose element already contains it. This ensures that, for each value read, the corresponding element of Interval has no more than *σ* letters added. As we consider at most *n* values, this step requires time and space $\mathcal {O}(n\sigma)$. For an example, see Figure 4.

#### Bottom-up pass

Intuitively, the idea behind the bottom-up pass is the same as in the top-down pass except that in this instance, as we start from the bottom, the suffix *y*[ SA[ *i*]..*n*−1] can share more than its prefix of length LCP[ *i*] with the previous suffixes in SA. Therefore we may need the elements of Interval that correspond to factors with an LCP value greater than LCP[ *i*] to correctly compute the arrays B_1_ and B_2_. To achieve this, we maintain another stack LifoRem to copy the values from LifoLCP that are greater than LCP[ *i*]. This extra stack allows us to keep in LifoLCP only values that are smaller or equal to LCP[ *i*] without losing the additional information we require to correctly compute B_1_ and B_2_. At the end of the iteration, we will set to zero each element corresponding to a value in LifoRem and empty the stack. Thus to set an element of Interval to zero requires two operations more than in the first pass. As we consider at most *n* values, this step requires time and space $\mathcal {O}(n\sigma)$.

**Figure 5:**
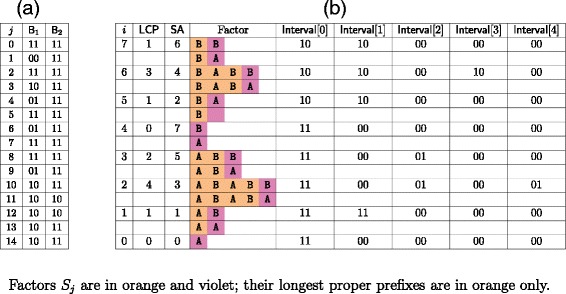
**Bottom-up pass.** **(a)** Arrays B_1_ and B_2_ obtained after the bottom-up pass for word *y*=AABABABB; **(b)** Elements of array Interval at the end of each iteration of the bottom-up pass.

Another difference between the top-down and bottom-up passes is that in order to retain the information computed in the first pass, the second step is performed for each letter in B_1_[ 2*i*]. As, for each LCP value read, we still add a letter only if is not already contained in the corresponding element of Interval, no more than *σ* letters are added. Thus this step requires time and space $\mathcal {O}(n\sigma)$. For an example, see Figure 5.

**Figure 6:**
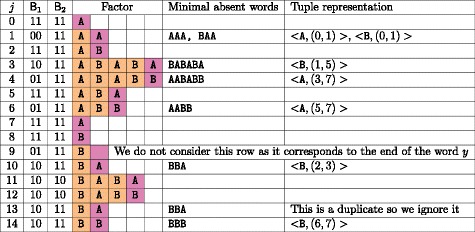
**Minimal absent words of word** ***y*** **=AABABABB; we find seven minimal absent words {AAA, AABABB, AABB, BAA, BABABA,BBA, BBB}.**

Once we have computed arrays B_1_ and B_2_, we need to compare each element. If there is a difference, by Lemma 1, we can construct a minimal absent word. For an example, see Figure 6. To ensure that we can report the minimal absent words in linear time, we must be able to report each one in constant time. To achieve this, we can represent them as a tuple <*a*,(*i*,*j*)>, where for some word *x* of length *m*≥2 that is a minimal absent word of *y*, the following holds: *x*[ 0]=*a* and *x*[ 1.. *m*−1]=*y*[ *i*.. *j*]. Note that this representation uniquely identifies a minimal absent word and conversion from this encoding to the actual minimal absent word is trivial. Lemma 2 ensures us to be exhaustive. Therefore we obtain the following result.

##### **Theorem1**.

Algorithm MAW solves problem MINIMALABSENTWORDS in time and space $\mathcal {O}(n)$.



## Results and discussion

The experiments were conducted on a Desktop PC using one core of Intel Xeon E5540 CPU at 2.5 GHz and 32GB of main memory under 64-bit GNU/Linux. All programs were compiled with gcc version 4.6.3 at optimisation level 3 (-O3). Time and memory measurements were taken using the GNU/Linux time command.

### Implementation

We implemented algorithm MAW as a program to compute all minimal absent words of a given sequence. The program was implemented in the C programming language and developed under GNU/Linux operating system. It takes as input arguments a file in (Multi)FASTA format and the minimal and maximal length of minimal absent words to be outputted; and then produces a file with all minimal absent words of length within this range as output. The implementation is distributed under the GNU General Public License (GPL), and it is available at http://github.com/solonas13/maw, which is set up for maintaining the source code and the man-page documentation.

### Datasets

We considered the genomes of thirteen bacteria and four case-study eukaryotes (Table 1), all obtained from the NCBI database (ftp://ftp.ncbi.nih.gov/genomes/).

**Table 1:** **Species selected for this work with reference to the respective abbreviation and identification of genome sequence data by accession number for bacteria or genome assembly project for eukaryotes**

**Species**	**Abbreviation**	**Genome reference**
**Bacteria**		
*Bacillus anthracis strain Ames*	Ba	NC003997
*Bacillus subtilis strain 168*	Bs	NC000964
*Escherichia coli strain K-12 substrain MG1655*	Ec	NC000913
*Haemophilus influenzae strain Rd KW20*	Hi	NC000907
*Helicobacter pylori strain 26695*	Hp	NC000915
*Lactobacillus casei strain BL23*	Lc	NC010999
*Lactococcus lactis strain Il1403*	Ll	NC002662
*Mycoplasma genitalium strain G37*	Mg	NC000908
*Staphylococcus aureus strain N315*	Sa	NC002745
*Streptococcus pneumoniae strain CGSP14*	Sp	NC010582
*Xanthomonas campestris strain 8004*	Xc	NC007086
**Eukaryotes**		
*Arabidopsis thaliana (thale cress)*	At	AGI release 7.2
*Drosophila melanogaster (fruit fly*)	Dm	FlyBase release 5
*Homo sapiens (human)*	Hs	build 38
*Mus musculus (mouse)*	Mm	build 38

### Correctness

To test the correctness of our implementation, we compared it against the implementation of Pinho et al. [6], which we denote here by PFG. In particular, we counted the number of minimal absent words, for lengths 11, 14, 17, and 24, in the genomes of the thirteen bacteria listed in Table 1. We considered only the 5^′^→3^′^ DNA strand. Table 2 depicts the number of minimal absent words in these sequences. We denote by M_11_, M_14_, M_17_, and M_24_ the size of the resulting sets of minimal absent words for lengths 11, 14, 17, and 24 respectively. Identical number of minimal absent words for these lengths were also reported by PFG, suggesting that our implementation is correct.

**Table 2:** **Number of minimal absent words of lengths 11, 14, 17, and 24 in the genomes of thirteen bacteria**

**Species**	**Genome size (bp)**	**M**_**11**_	**M**_**14**_	**M**_**17**_	**M**_**24**_
Ba	5,227,293	1,113,398	1,001,357	32,432	46
Bs	4,214,630	951,273	1,703,309	86,372	226
Ec	4,639,675	1,072,074	1,125,653	36,395	247
Hi	1,830,023	722,860	294,353	12,158	91
Hp	1,667,825	564,308	336,122	19,276	75
Lc	3,079,196	1,126,363	502,861	13,083	246
Ll	2,365,589	764,006	507,490	25,667	183
Mg	1,664,957	246,342	66,324	2,737	28
Sa	2,814,816	755,483	704,147	32,054	138
Sp	2,209,198	904,815	327,713	10,390	234
Xc	5,148,708	804,034	1,746,214	179,346	633

### Efficiency

**Figure 7:**
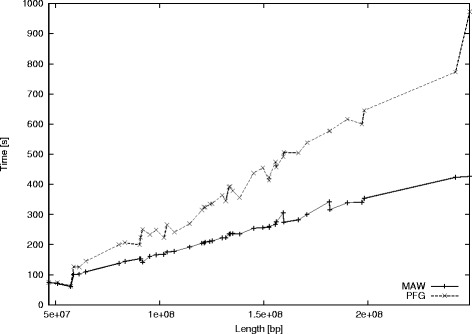
**Chromosome sequence length versus elapsed time for computing all minimal absent words in the genomes of** ***Homo Sapiens*** **and** ***Mus musculus*** **using** **MAW** **and** **PFG** **.**

To evaluate the efficiency of our implementation, we compared it against the corresponding performance of PFG, which is currently the fastest available implementation for computing minimal absent words. Notice that this evaluation depends heavily on the suffix array construction implementation used; and that PFG uses a less optimised implementation for this construction than the one used by MAW. We computed all minimal absent words for each chromosome sequence of the genomes of the four eukaryotes listed in Table 1. We considered both the 5^′^→3^′^ and the 3^′^→5^′^ DNA strands. Tables 3 and 4 depict elapsed-time comparisons of MAW and PFG. We observe that PFG scales mostly linearly. MAW also scales linearly and is the fastest in *all* cases. It accelerates the computations by more than a factor of 2, when the sequence length grows, compared to PFG. Figure 7 corresponds to the measurements in Table 4: it plots chromosome sequence length versus elapsed time for computing all minimal absent words in the genomes of *Homo Sapiens* and *Mus musculus* using MAW and PFG. MAW also reduces the memory requirements by a factor of 5 compared to PFG. The maximum allocated memory (per task) was 6GB for MAW and 30GB for PFG.

**Table 3:** **Elapsed-time comparison of** **MAW** **and** **PFG** **for computing all minimal absent words in the genome of** ***Arabidopsis thaliana*** **and** ***Drosophila melanogaster***

**(a) At**			
**Chromosome**	**Size (bp)**	**MAW** ** (s)**	**PFG** ** (s)**
1	30,427,671	40.20	51.90
2	19,698,289	25.86	32.94
3	23,459,830	30.84	42.30
4	18,585,056	24.65	31.42
5	26,975,502	35.38	48.91
**(b) Dm**			
**Chromosome**	**Size (bp)**	**MAW** ** (s)**	**PFG** ** (s)**
2L	23,011,544	30.01	40.85
2R	21,146,708	27.52	38.38
3L	24,543,557	32.00	45.13
3R	27,905,053	36.44	48.36
X	22,422,827	29.38	40.09

**Table 4:** **Elapsed-time comparison of** **MAW** **and** **PFG** **for computing all minimal absent words in the genome of** ***Homo Sapiens*** **and** ***Mus musculus***

**(a) Hs**			
**Chromosome**	**Size (bp)**	**MAW** ** (s)**	**PFG** ** (s)**
1	248,956,422	426.39	972.52
2	242,193,529	423.19	772.89
3	198,295,559	353.60	645.45
4	190,214,555	339.02	616.26
5	181,538,259	342.53	577.05
6	170,805,979	299.72	538.34
7	159,345,973	305.26	491.32
8	145,138,636	254.17	437.18
9	138,394,717	235.14	356.08
10	133,797,422	235.38	392.45
11	135,086,622	236.80	379.15
12	133,275,309	235.14	390.46
13	114,364,328	191.64	269.52
14	107,043,718	178.00	240.93
15	101,991,189	167.89	222.98
16	90,338,345	153.07	198.49
17	83,257,441	144.32	207.02
18	80,373,285	137.68	199.44
19	58,617,616	100.95	126.82
20	64,444,167	109.80	144.83
21	46,709,983	74.60	74.65
22	50,818,468	70.49	73.34
X	156,040,895	275.14	457.2
Y	57,227,415	60.85	62.34
**(b) Mm**			
**Chromosome**	**Size (bp)**	**MAW** ** (s)**	**PFG** ** (s)**
1	197,195,432	340.59	599.86
2	181,748,087	316.17	578.2
3	159,599,783	274.46	506.73
4	155,630,120	266.67	473.97
5	152,537,259	260.50	424.24
6	149,517,037	256.36	455.11
7	152,524,553	257.65	413.37
8	131,738,871	223.09	344.92
9	124,076,172	210.37	334.25
10	129,993,255	222.36	363.34
11	121,843,856	208.55	324.54
12	121,257,530	205.09	324.79
13	120,284,312	204.80	314.56
14	125,194,864	212.59	336.49
15	103,494,974	175.21	265.92
16	98,319,150	166.10	249.03
17	95,272,651	160.70	232.79
18	90,772,031	153.40	223.56
19	61,342,430	101.89	125.85
X	166,650,296	282.21	503.98
Y	91,744,698	141.79	251

To further evaluate the efficiency of our implementation, we compared it against the corresponding performance of PFG using synthetic data. As basic dataset we used chromosome 1 of Hs. We created five instances S_1_, S_2_, S_3_, S_4_, and S_5_ of this sequence by randomly choosing 10%, 20%, 30%, 40%, and 50% of the positions, respectively, and randomly replacing the corresponding letters to one of the four letters of the DNA alphabet. We computed all minimal absent words for each instance. We considered both the 5^′^→3^′^ and the 3^′^→5^′^ DNA strands. Table 5 depicts elapsed-time comparisons of MAW and PFG. MAW is the fastest in *all* cases.

**Table 5:** **Elapsed-time comparison of** **MAW** **and** **PFG** **for computing all minimal absent words in synthetic data**

**Sequence**	**Size (bp)**	**MAW** ** (s)**	**PFG** ** (s)**
S_1_	248,956,422	435.63	746.93
S_2_	248,956,422	438.52	733.69
S_3_	248,956,422	444.62	726.34
S_4_	248,956,422	444.06	743.29
S_5_	248,956,422	449.25	741.01

## Conclusions

In this article, we presented the first $\mathcal {O}(n)$-time and $\mathcal {O}(n)$-space algorithm for computing all minimal absent words based on the construction of suffix arrays. In addition, we provided the respective implementation of this algorithm. Experimental results show that this implementation outperforms existing tools, both in terms of speed and memory.

In a typical application, one would be interested in computing minimal absent words in the whole genome for a set of species under study [11,12]. Hence, we consider the improvements described in this article to be of great importance. Our immediate target is twofold: first, explore the possibility of implementing the presented algorithm for symmetric multiprocessing systems; and second, devise and implement a fast space-efficient solution for this problem based on the construction of compressed full-text indexes.

## Availability and requirements

- **Project name:**MAW
- **Project home page:**http://github.com/solonas13/maw
- **Operating system:** GNU/Linux
- **Programming language:** C
- **Other requirements:** compiler gcc version 4.6.3 or higher
- **License:** GNU GPL
- **Any restrictions to use by non-academics:** licence needed
